# Supplementary material for: Predicted distribution of a rare and understudied forest carnivore: Humboldt marten (Martes caurina humboldtensis)
Source: PeerJ. 2021 Jul 21;9:e11670. doi: 10.7717/peerj.11670 (PMC8354145; doi:10.7717/peerj.11670)
Supplement: Supplemental Information 1 — We describe the old growth structural index in detail and evaluate Humboldt marten (Martes caurina humboldtensis) locations in relation to existing models and each of the remotely sensed vegetative components. We provide boxplots, histograms, and results from two additional Maxent models. [file peerj-09-11670-s001.pdf]

Supplemental Item S1. Investigation of the variable "old growth structural index" as a surrogate for Humboldt marten habitat: An exploration of inconsistencies between two range-wide habitat models.

Supplemental item for the manuscript:

Predicted distribution of a rare and understudied forest carnivore: Humboldt martens (*Martes caurina humboldtensis*)

Katie Moriarty[1], Joel Thompson[2], Matthew Delheimer[3], Brent Barry[4], Mark Linnell[5], Taal Levi[6], Keith Hamm[7], Desiree Early[7], Holly Gamblin[8], Micaela Szykman Gunther[8], Jordan Ellison[1], Janet S. Prevéy[9], Jennifer Hartman[10], Ray Davis[11]

Here, we provide a detailed evaluation and comparison of the results of our range-wide Humboldt marten (*Martes caurina humboldtensis*) habitat model, presented within the main manuscript, with a previously-published model (Slauson et al. 2019). In particular, the Slauson et al. (2019) model places substantial emphasis on Humboldt marten occurrence being strongly and positively associated with an "old growth structural index" variable (hereafter, OGSI), yet we found little evidence for a similar relationship within our model. Given that OGSI is already being used as a "surrogate" for Humboldt marten habitat (e.g., Schrott and Shinn 2020, Supplemental Item Fig. S1), it may behoove managers and wildlife practitioners to understand the differences between variables incorporated into our respective models and their influences on subsequent model outputs.

What is the old growth structural index and how is it calculated?

OGSI is a composite index that combines several spatially-explicit, remotely-derived forest structure elements using Lemma's gradient nearest neighbor index (Ohmann and Gregory 2002). OGSI was designed to describe the continuum of forest succession, with higher values in the later stages of succession (Spies and Franklin 1988). OGSI extends in geography to Washington, Oregon, and California and was created in part to monitor old forest conditions over broad spatial scales (Davis et al. 2015), especially areas within the Northwest Forest Plan and over the range of the northern spotted owl (*Strix occidentalis caurina*) (Davis et al. 2016).

As an index, OGSI has evolved in both complexity and precision over time. For example, the 2006 version was calculated from five elements, including: tree age (age_dom); density of large live trees (>100 cm in diameter; tph_ge_100cm dbh); a diameter diversity index computed from tree densities in different diameter classes (ddi); density of large snags (stph_5015); and percentage of downed wood greater than 25cm in diameter (dvph_ge_25). The 2006 version of OGSI had the same inputs for all vegetative zones in the Pacific Northwest (see code block below for more detail) and index values ranged from 0 to 100. Since 2010, OGSI has been calculated from four elements: density of large live trees per hectare (ltphc); density of large snags per hectare (stph_ge); percentage of downed wood greater than 25cm in diameter (dcov_ge_25cm); and an index of diversity of tree diameter computed from tree densities in different diameter classes (ddi). Unlike the 2006 version, the more recent version has twelve vegetative zones that each have a unique threshold for what is

considered a “large” live tree or a “large” snag ranging from 50 to 100cm for live trees and 50 to 75cm for calculating snag densities. In other words, this metric is dependent on forest type – for example, a “large diameter” tree or snag in a lodgepole pine (*Pinus contorta*) stand would be considered comparatively “small diameter” within a coastal redwood (*Sequoia sempervirens*) stand. Similar to the 2006 version, the 2010 OGSI version ranges between 0 and 100 (see Davis et al. 2015 and the infographic on page 6).

Why use the old growth structural index?

With too much data or too few replicates, condensing variables into a composite index such as OGSI can be a useful tool for modeling. There are both formal and practical procedures for creating such indices. Formal methods are often applied, for example, if a goal is to describe vegetation associations with hundreds of variables (e.g., canopy cover, number and diameter of each tree species, stems per shrub, leaves per shrub, etc.), because a model would be computationally intractable with too many variables. In wildlife, common opportunities to reduce many variables into two or three composite variables include principal component, generalized discriminant, and canonical correlation analyses (Ramsey and Schafer 2002). Similarly, but less formally, one can combine correlated variables with *a priori* hypotheses or biological logic by adding the values. The challenge of interpreting such data is that the results are an index, not a feature. For instance, instead of describing the relationship of Humboldt marten locations to canopy cover, one would describe the relationship between “axis 1”

and “axis 2” or an index without defining which components are most related to the species of interest.

The challenge of index interpretation and relating it to biological expectations is not unique to OGSI. Similarly and more simply in wildlife habitat relationships, quadradic mean diameter (QMD) is $\sqrt{(\sum d_i^2)/\mathrm{n}}$ where *d* is the diameter of an individual (*i*) tree at breast height and n is the number of trees (Curtis and Marshall 2000). QMD has been used in silviculture since the early 1900s (e.g., Graves 1908) and is one of the primary components in the California Wildlife Habitat Relationship database to assign a habitat value (e.g., high or low quality) to a location based on vegetation elements (Salwasser and Laudenslayer 1982, Garrison 1994). By the nature of the calculation, the QMD value of a given forest stand increases when it is thinned, as the result of the removal of small diameter trees (Curtis and Marshall 2000). While habitat quality is generally presumed to improve with increasing QMD values for many forest-dependent species such as Pacific martens, processes such as forest thinning may in fact degrade habitat quality (e.g., Stephens et al. 2014, Moriarty et al. 2016) despite the appearance of improvement (i.e., increased QMD). As such, interpretation of indices such as OGSI or QMD can be challenging and not associated with biological realities if the situational components are not clearly described.

Humboldt marten locations and OGSI

We modeled using the 2016 version of the OGSI variable, despite its meager contributions to our model iterations (<5% contribution), primarily for purposes of comparison with the Slauson et al. (2019) model. When incorporated into our model, the

relationship between OGSI and Humboldt marten locations was not only weak but also often negative – higher OGSI values could be interpreted as less suitable for predicted Humboldt marten use.

We assume that both modeling efforts used the best available data, but the amount of effort and geographic scope of surveys for Humboldt martens have exponentially increased since 2010, the last year that data were considered for the Slauson et al. (2019) model. Although their text describes 1,159 considered surveys, their model used 559 non-detection and 44 detection locations (Table 5), with detection data being strongly spatially autocorrelated (e.g., Slauson et al. 2019, Fig. 5). Detections occurred primarily in northern California (n = 36 detections, 82%) and included a relatively small number of locations from Oregon (n = 8 detections, 18%). Further, much of the survey effort considered for the Slauson et al. (2019) model was intended to detect fishers (*Pekania pennanti;* e.g., Carroll et al. 1999, Zielinski et al. 2010), which are larger-bodied and have substantially larger home ranges than martens. The spacing of such efforts compared to surveys intended for martens – approximately 6 km between survey points versus approximately 2 km between points – may have occurred at too coarse of a scale to detect martens, with their smaller home ranges and rigid territoriality (Moriarty et al. 2017).

In our model, we combined efforts from various studies specifically designed to survey for Humboldt martens (Slauson et al. 2007, Barry 2018, Linnell et al. 2018, Moriarty et al. 2018, Gamblin 2019, Moriarty et al. 2019) while also including data used in the Slauson et al. (2019) model. Surveys included in our model had broad-scale coverage in Oregon and were randomly or evenly-distributed throughout the entire

coast range, including all forested age classes (Moriarty et al. 2018, Moriarty et al. 2019). We modeled using a relatively even proportion of locations throughout the range of the Humboldt marten in both California and Oregon, including data from areas previously identified as unsuitable (e.g., Zielinski et al. 2001). We compiled 10,229 locations (6,768 detections, 3,461 non-detections) from 1996-2020, thinned the data to one location within a 500m by 500m cell, and modeled based on 384 locations (see main manuscript for details).

Using our expanded location dataset, we investigated the assumption that Humboldt marten occurrence is strongly associated with increasing OGSI values, using summary data and models. A histogram of marten location data did not immediately suggest that there were more marten locations with increased OGSI values (Supplemental Item Fig. S3). Similarly, our thinned Humboldt marten locations were not extremely different from random locations at any measured spatial scale (Supplemental Item Fig. S4). Given that OGSI is a composite index, we further investigated the influence of the OGSI variable relative to the influence of each component variable and recreated the index as it would have been used in the 2006 version with 5 variables (forest age, diameter diversity index, large snag density, large tree density, and downed wood density). We deconstructing the OGSI variable, and modeled Humboldt marten distribution using only OGSI or including the five component variables without additional co-variates. Our model with OGSI as the sole variable to evaluate Humboldt marten distribution performed similar to a random variable (Supplemental Item Fig. S5). Our Humboldt marten model with each of the five OGSI components did better in creating a more interpretable map, possibly because the response curves could vary

(Supplemental Item Fig. S6). Here, the variables that explained the most variation were percentage of downed wood and the diameter diversity index (Supplemental Item Table S1).

The weak contribution of OGSI to our model suggests that Slauson et al. (2019) may have overemphasized the importance of OGSI and underestimated the implications of abiotic factors. Within Slauson et al. (2019), the top-ranked model included four variables: (1) OGSI at a 1km scale; (2) serpentine at a 3km scale; (3) precipitation; and (4) adjusted elevation. Given the limitations of the dataset incorporated into the Slauson et al. (2019) model – specifically, incorporation of a small number of detections and poor coverage across the full putative distribution on the Humboldt marten – the determination that the OGSI variable was strongly influential across the entire range of the Humboldt marten may also have been an interpretive extrapolation beyond the scope of their data. Similar to the functional response curve within the previous model (Slauson et al. 2019, Fig. 3), we observed a generally neutral or negative relationship of Humboldt marten locations and OGSI with our univariate response curves.

Although Humboldt marten locations appear to be weakly and potentially negatively associated with OGSI, we are not suggesting that Humboldt martens avoid older structures with complex features such as cavities or mistletoe (Slauson and Zielinski 2009, Tweedy et al. 2019). Such individual structures and microsites are strongly linked to resting and denning in Pacific martens and fishers (e.g., Matthews et al. 2019, Tweedy et al. 2019). Nonetheless, Humboldt marten locations and predicted habitat appear variable in relation to vegetation characteristics. While factors such as

OGSI may be correlated to Humboldt marten locations at a local or regional level (e.g., in portions of northern California), based on available data, it is inappropriate to use OGSI as a surrogate for predicted habitat throughout the Humboldt marten range. Regardless, habitat models are an evolving opportunity to learn and we applaud efforts to continue data collection, address challenging information gaps, and inform conversation efforts.

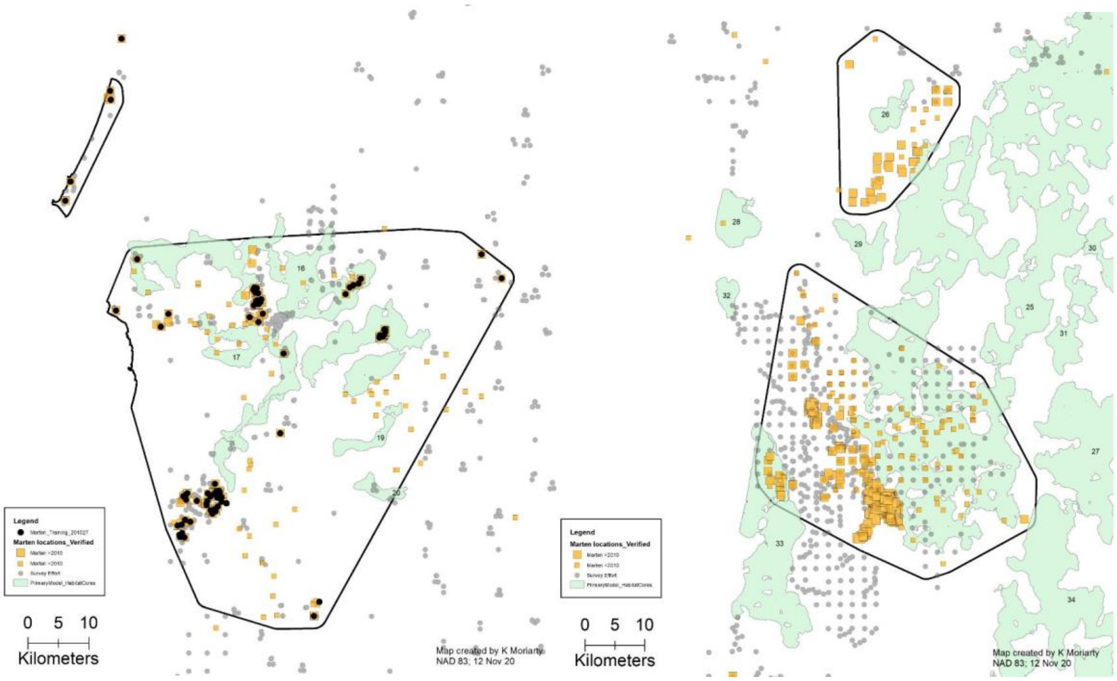

Supplemental Item Figure S1. Prior models use a remotely sensed variable, old growth structural index (OGSI), to depict “habitat cores” (Schrott and Shinn 2020). Here, we provide examples of those cores (light green) with survey detection/non-detection locations focused on Humboldt marten distribution (orange). Black lines are areas Humboldt marten population designations and grey icons were surveyed but did not detect a marten. The green polygons are used habitat cores within the USFWS connectivity model (Schrott and Shinn 2020), containing ~9% of known Humboldt marten locations.

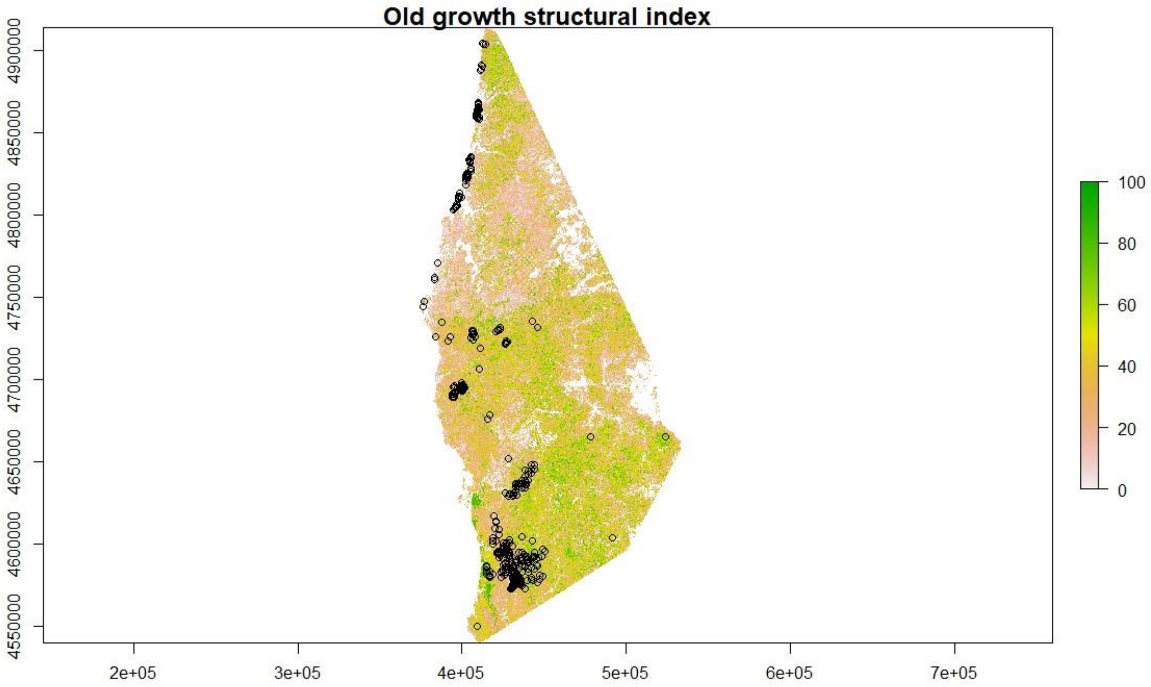

Supplemental Item Figure S2. We display the 2016 remotely sensed index old growth structural index (OGSI) distribution within the current extent of Humboldt marten (*Martes caurina humboldtensis*) locations. High values of OGSI are green. Humboldt marten locations are black outlined dots.

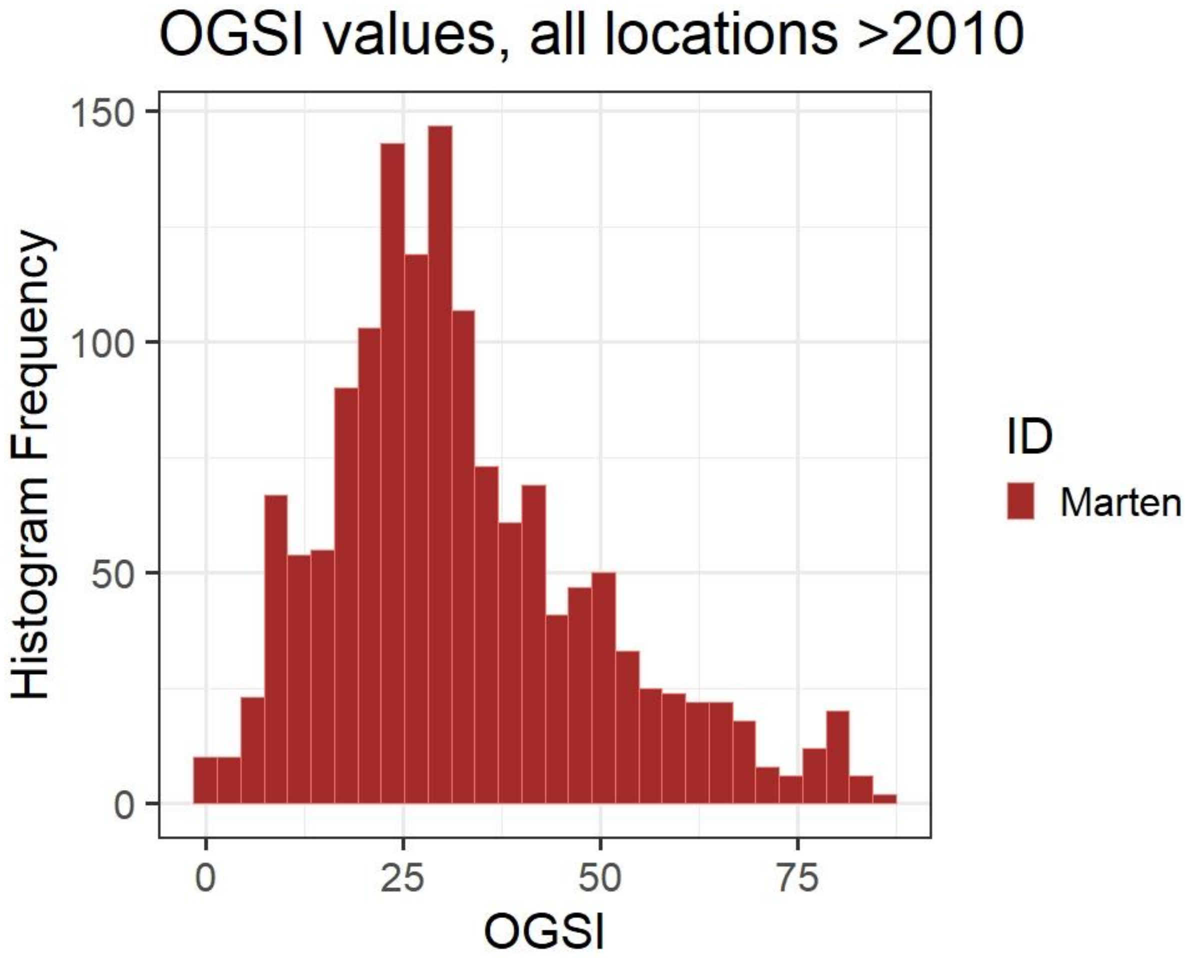

Supplemental Item Figure S3. Histogram of the remotely sensed index old growth structural index (OGSI) and the value for all known Humboldt marten locations. The median value of OGSI within the historic Humboldt marten range with the 2012 vegetation layer was an index of 36 (Schrott and Shinn 2020). Here, notice the majority of marten locations were located in areas with OGSI values less than 36.

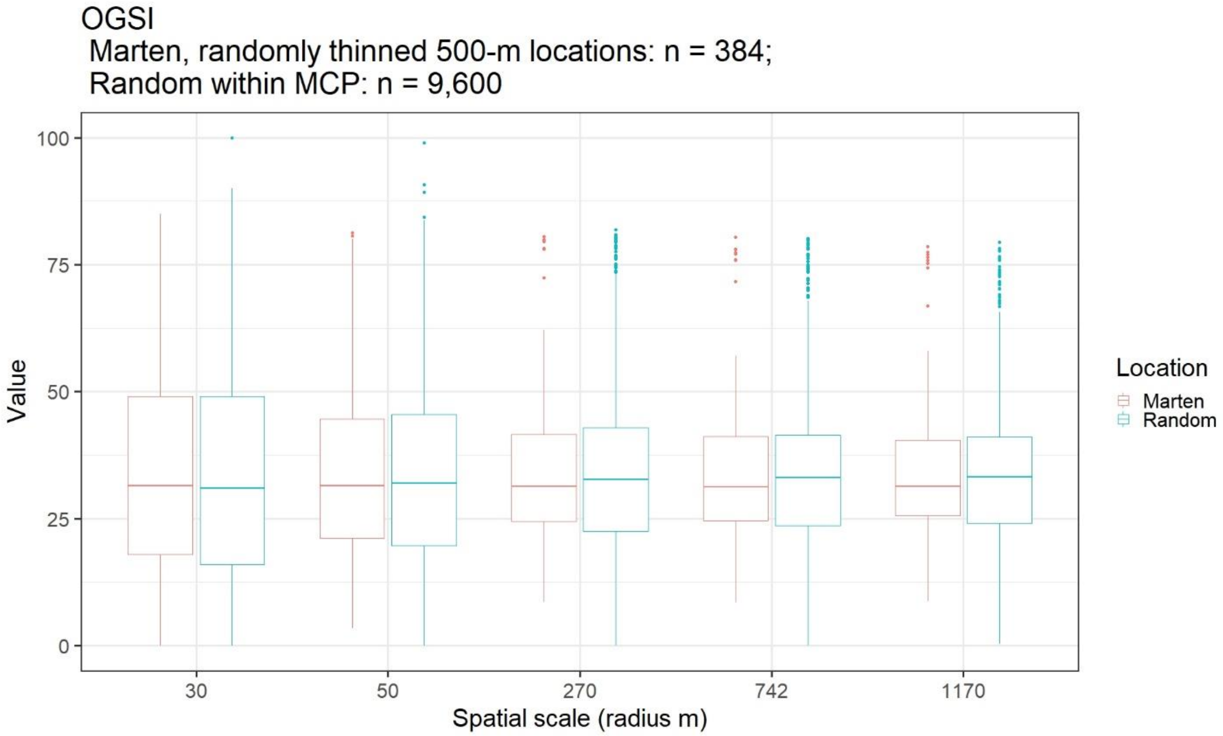

Supplemental Item Figure S4. We compared the spatially thinned location data with 25 random locations per known (9,600) at spatial scales presumed relevant to Humboldt marten biology. The median for Humboldt marten locations and random locations was similar at each spatial scale. With a focal radius >30m, the median for random values is slightly higher than marten locations. Univariate generalized linear model beta coefficients using these data starting at 50m were 0.0014, 0.00028, -0.00094, and -0.00249, respectively. These suggest that when averaging at large spatial scales (742m, 1170m) the relationship between marten locations and OGSI were negative.

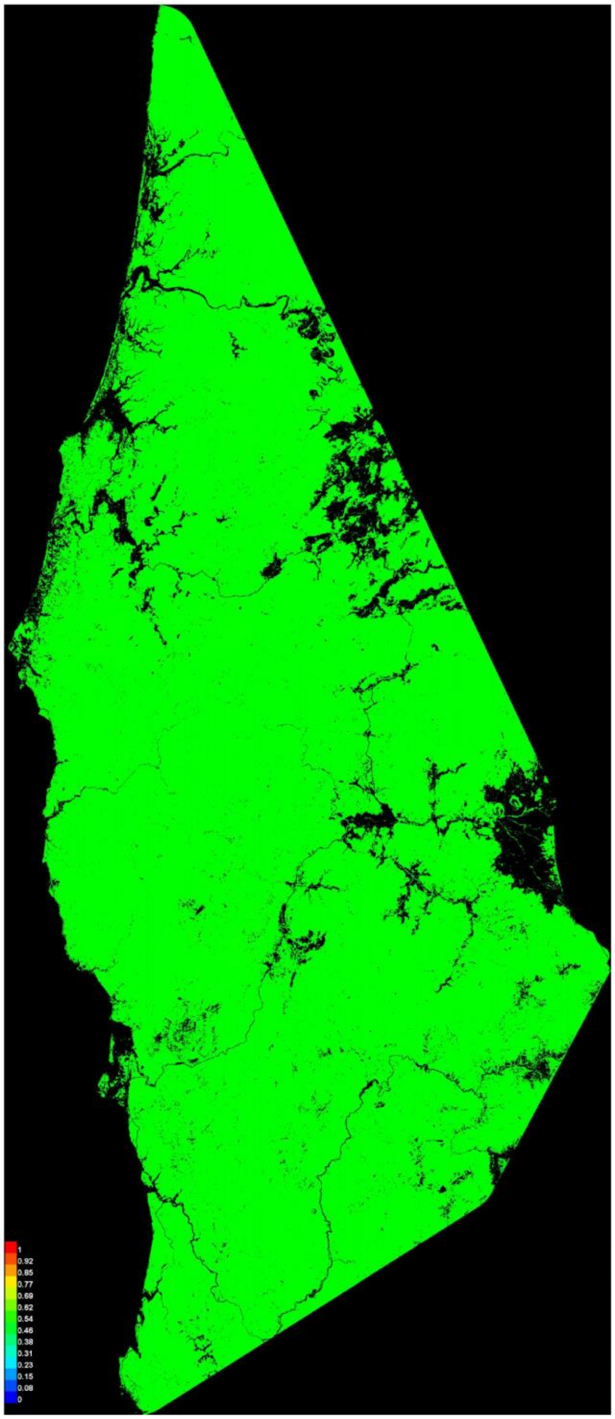

Supplemental Item Figure S5. We created a Maxent model only with the variable old growth structural index (OGSI). Here, it predicted Humboldt marten (*Martes caurina humboldtensis*) distribution slightly above a random value. Green is approximately 50% predicted probability and red would indicate high correlation with Humboldt marten locations.

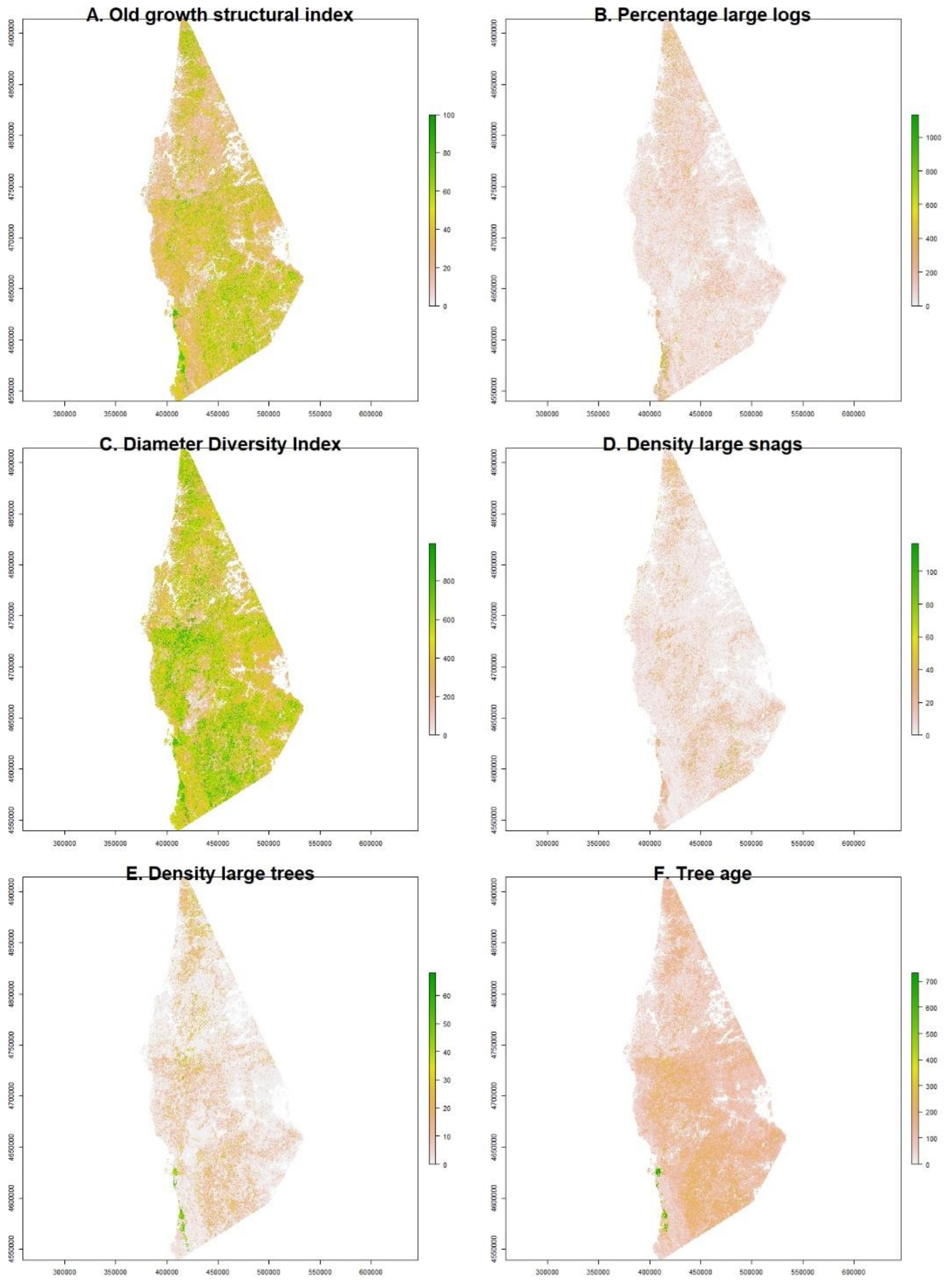

Supplemental Item Figure S6. We separated the index OGSI (A) to each of its components to investigate which element(s) within the OGSI index were correlated with Humboldt martens. The 5 components of OGSI, similar to the 2006 version, include percentage of large logs (B), Diameter Diversity Index (C), Density of large snags (D), Density of large trees (E), and Tree age (F). These 5 components were ordered in relation to predicted probability of Humboldt marten (*Martes caurina humboldtensis*) occurrence (Supplemental Item Table S1).

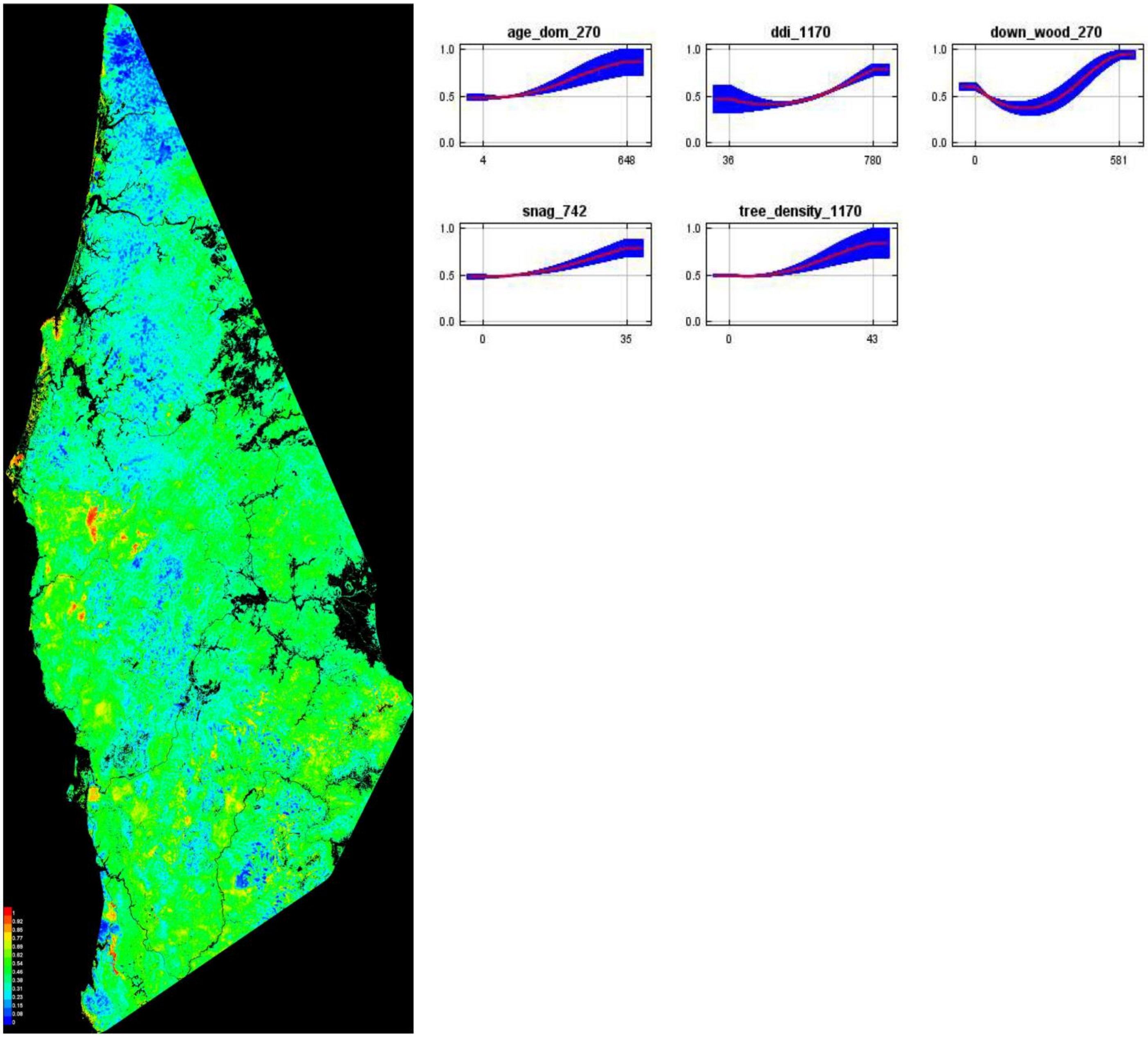

Supplemental Item Figure S7. We depict a spatial map of predicted Humboldt marten range from a Maxent model using the 5 components of the variable old growth structural index (OGSI), with our known and thinned Humboldt marten occurrences (n = 384). From these components, percentage of downed wood at a smoothed radius of 270m (down_wood_270), diameter diversity index at a smoothed radius of 1170m scale (ddi_1170), large tree density (tree_density_1170), large snag density (snag_742) and estimated tree age (age_dom_270) were the order of model rank by percent contribution.

Supplemental Item Table S1. We created a Maxent model using the 5 components of the variable old growth structural index (OGSI). When evaluating either percent contribution or permutation importance, estimated tree age contributed least and either downed wood or diameter diversity contributed most to the predicted model.

Variable	Scale	Relationship	Percent contribution	Permutation importance
Downed wood	270	+	36	21.2
Diameter diversity index	1170	+	23.5	36.2
Large tree density	1170	+	19.2	15.9
Large snag density	742	+	13.7	14.5
Age dominant forest	270	+	7.5	12.2

Literature Cited

Barry, B. R. 2018. Distribution, habitat associations, and conservation status of Pacific fisher (*Pekania pennanti*) in Oregon. Oregon State University, Corvallis, Oregon, USA.

Bell, D. M., S. A. Acker, M. J. Gregory, R. J. Davis, and B. A. Garcia. 2021. Quantifying regional trends in large live tree and snag availability in support of forest management. Forest Ecology and Management 479:118554.

Carroll, C. R., W. J. Zielinski, and R. F. Noss. 1999. Using presence-absence data to build and test spatial habitat models for the fisher in the Klamath Region, USA. Conservation Biology 13:1344-1359.

Curtis, R. O., and D. D. Marshall. 2000. Technical note: why quadratic mean diameter? Western Journal of Applied Forestry 15:137-139.

Davis, R., J. Ohmann, R. Kennedy, W. Cohen, M. Gregory, Z. Yang, H. Roberts, A. Gray, and T. Spies. 2015. Northwest Forest Plan – the first 20 years (1994-2013): status and trends of late-successional and old-growth forests *in* USDA Forest Service: Portland, OR, USA.

Davis, R. J., B. Hollen, J. Hobson, J. E. Gower, and D. Keenum. 2016. Northwest Forest Plan—the first 20 years (1994–2013): status and trends of northern spotted owl habitats. U.S. Department of Agriculture, Forest Service, Pacific Northwest Research Station. General Technical Report PNW-GTR-929.

Gamblin, H. E. 2019. Distribution and habitat use of a recently discovered population of Humboldt martens in California. Humboldt State University, Arcata, CA, USA.

Garrison, B. A. 1994. Determining the biological significance of changes in predicted habitat values from the California Wildlife Habitat Relationships System. California Fish and Game 80:150-160.

Graves, H. S. 1908. Forest mensuration. Wiley Press, New York, United States.

Green, R. 2017. Reproductive ecology of the fisher (Pekania pennanti) in the southern Sierra Nevada: an assessment of reproductive paramaters and forest habitat used by denning females. Dissertation, University of California, Davis, CA, USA.

Green, R. E., K. L. Purcell, C. M. Thompson, D. A. Kelt, and H. U. Wittmer. 2019. Microsites and structures used by fishers (Pekania pennanti) in the southern Sierra Nevada: A comparison of forest elements used for daily resting relative to reproduction. Forest Ecology and Management 440:131-146.

Joyce, M. J., J. D. Erb, B. A. Sampson, and R. A. Moen. 2019. Detection of coarse woody debris using airborne light detection and ranging (LiDAR). Forest Ecology and Management 433:678-689.

Kerns, B. K., and J. L. Ohmann. 2004. Evaluation and prediction of shrub cover in coastal Oregon forests (USA). Ecological Indicators 4:83-98.

Linnell, M. A., K. Moriarty, D. S. Green, and T. Levi. 2018. Density and population viability of coastal marten: a rare and geographically isolated small carnivore. PeerJ 6:e4530 - '4521 pg.

Matthews, S. M., D. S. Green, J. M. Higley, K. M. Rennie, C. M. Kelsey, and R. E. Green. 2019. Reproductive den selection and its consequences for fisher neonates, a cavity-obligate mustelid. Journal of Mammalogy 100:1305-1316.

Moriarty, K. M., C. W. Epps, and W. J. Zielinski. 2016. Forest thinning for fuel reduction changes movement patterns and habitat use by Pacific marten. The Journal of Wildlife Management 80:621-633.

Moriarty, K. M., M. A. Linnell, B. Chasco, C. W. Epps, and W. J. Zielinski. 2017. Using high-resolution short-term location data to describe territoriality in Pacific martens. Journal of Mammalogy 98:679-689.

Moriarty, K. M., M. A. Linnell, J. E. Thornton, and G. W. Watts III. 2018. Seeking efficiency with carnivore survey methods: a case study with elusive martens. Wildlife Society Bulletin 42:403-413.

Moriarty, K. M., J. Verschuyl, A. J. Kroll, R. Davis, J. Chapman, and B. Hollen. 2019. Describing vegetation characteristics used by two rare forest-dwelling species: Will established reserves provide for coastal marten in Oregon? PLoS ONE 14:e0210865.

Ohmann, J. L., and M. J. Gregory. 2002. Predictive mapping of forest composition and structure with direct gradient analysis and nearest-neighbor imputation in coastal Oregon, USA. Canadian Journal of Forest Research 32:725-741.

Ramsey, F. L., and D. W. Schafer. 2002. The statistical sleuth: a course in the methods of data analysis. Second edition. Duxbury, Pacific Grove, California, USA.

Salwasser, H., and W. F. Laudenslayer. 1982. The California wildlife/fish habitat relationships system. Transactions of the Western Section of the Wildlife Society 18:27-33.

Schrott, G. R., and J. Shinn. 2020. A landscape connectivity analysis for the coastal marten (*Martes caurina humboldtensis*). USDI Fish and Wildlife Service.

Slauson, K. M., and W. J. Zielinski. 2009. Characteristics of summer and fall diurnal resting habitat used by American martens in coastal northwestern California. Northwest Science 83:35-45.

Slauson, K. M., W. J. Zielinski, and J. P. Hayes. 2007. Habitat selection by American martens in coastal California. Journal of Wildlife Management 71:458-468.

Slauson, K. M., W. J. Zielinski, D. W. LaPlante, and T. A. Kirk. 2019. A landscape suitability model for the Humboldt marten (*Martes caurina humboldtensis*) in coastal California and coastal Oregon. Northwest Science.

Spies, T. A., and J. F. Franklin. 1988. Old growth and forest dynamics in the Douglas-fir region of western Oregon and Washington. Natural Areas Journal 8:190-201.

Stephens, S. L., S. W. Bigelow, R. D. Burnett, B. M. Collins, C. V. Gallagher, J. Keane, D. A. Kelt, M. P. North, L. J. Roberts, and P. A. Stine. 2014. California spotted owl, songbird, and small mammal responses to landscape fuel treatments. BioScience.

Tweedy, P. J., K. M. Moriarty, J. D. Bailey, and C. W. Epps. 2019. Using fine scale resolution vegetation data from LiDAR and ground-based sampling to predict Pacific marten resting habitat at multiple spatial scales. Forest Ecology and Management 452:117556.

Wing, B. M., M. W. Ritchie, K. Boston, W. B. Cohen, and M. J. Olsen. 2015. Individual snag detection using neighborhood attribute filtered airborne lidar data. Remote Sensing of Environment 163:165-179.

Zielinski, W. J., J. R. Dunk, J. S. Yaeger, and D. W. LaPlante. 2010. Developing and testing a landscape-scale habitat suitability model for fisher (*Martes pennanti*) in forests of interior northern California. Forest Ecology and Management 260:1579-1591.

Zielinski, W. J., K. M. Slauson, C. R. Carroll, C. J. Kent, and D. G. Kudrna. 2001. Status of American martens in coastal forests of the Pacific states. Journal of Mammalogy 82:478-490.

Code for 2006 OGSI index:

```
CREATE FUNCTION dbo.GET_OGSI
	(@age_dom DECIMAL(9,4), @tph_ge_100 DECIMAL(9,4),
	 @ddi DECIMAL(9,4), @stph_5015 DECIMAL(9,4), @dvph_ge_25
DECIMAL(9,4))
RETURNS DECIMAL(9,4) AS

BEGIN

	DECLARE @age_score FLOAT, @tph_score FLOAT
	DECLARE @ddi_score FLOAT, @snag_score FLOAT
	DECLARE @cwd_score FLOAT, @ogsi DECIMAL(9,4)

	--Live tree age
	IF @age_dom <= 200.0
		SET @age_score = 0.004 * @age_dom
	ELSE IF @age_dom > 200.0 AND @age_dom <= 450.0
		SET @age_score = 0.64 + (0.0008 * @age_dom)
	ELSE IF @age_dom > 450
		SET @age_score = 1.0

	--Live TPH
	IF @tph_ge_100 <= 17.0
		SET @tph_score = 0.02941 * @tph_ge_100
	ELSE IF @tph_ge_100 > 17.0 AND @tph_ge_100 <= 32.0
		SET @tph_score = 0.21667 + (0.01667 * @tph_ge_100)
	ELSE IF @tph_ge_100 > 32.0 AND @tph_ge_100 <= 55.0
		SET @tph_score = 0.40217 + (0.01087 * @tph_ge_100)
	ELSE
		SET @tph_score = 1.0

	--Diameter diversity index
	SET @ddi_score = 0.1 * @ddi

	--Snag TPH
	IF @stph_5015 <= 1.0
		SET @snag_score = 0.5 * @stph_5015
```

```
	ELSE IF @stph_5015 > 1.0 AND @stph_5015 <= 3.0
		SET @snag_score = 0.375 + (0.125 * @stph_5015)
	ELSE IF @stph_5015 > 3.0 AND @stph_5015 <= 14.0
		SET @snag_score = 0.68182 + (0.02273 * @stph_5015)
	ELSE
		SET @snag_score = 1.0

	--Coarse woody debris volume
	IF @dvph_ge_25 <= 40.0
		SET @cwd_score = 0.0125 * @dvph_ge_25
	ELSE IF @dvph_ge_25 > 40.0 AND @dvph_ge_25 <= 260.0
		SET @cwd_score = 0.45455 + (0.00114 * @dvph_ge_25)
	ELSE IF @dvph_ge_25 > 260.0 AND @dvph_ge_25 <= 630.0
		SET @cwd_score = 0.57432 + (0.00067568 * @dvph_ge_25)
	ELSE
		SET @cwd_score = 1.0

	--Composite old growth habitat index
	SET @ogsi =
		((@age_score + @tph_score + @ddi_score + @snag_score +
@cwd_score)/5.0)*100.0

	RETURN @ogsi

END
```
