# Supplementary figures and images for: Predicted distribution of a rare and understudied forest carnivore: Humboldt marten (Martes caurina humboldtensis)
Source: PeerJ. 2021 Jul 21;9:e11670. doi: 10.7717/peerj.11670 (PMC8354145; doi:10.7717/peerj.11670)

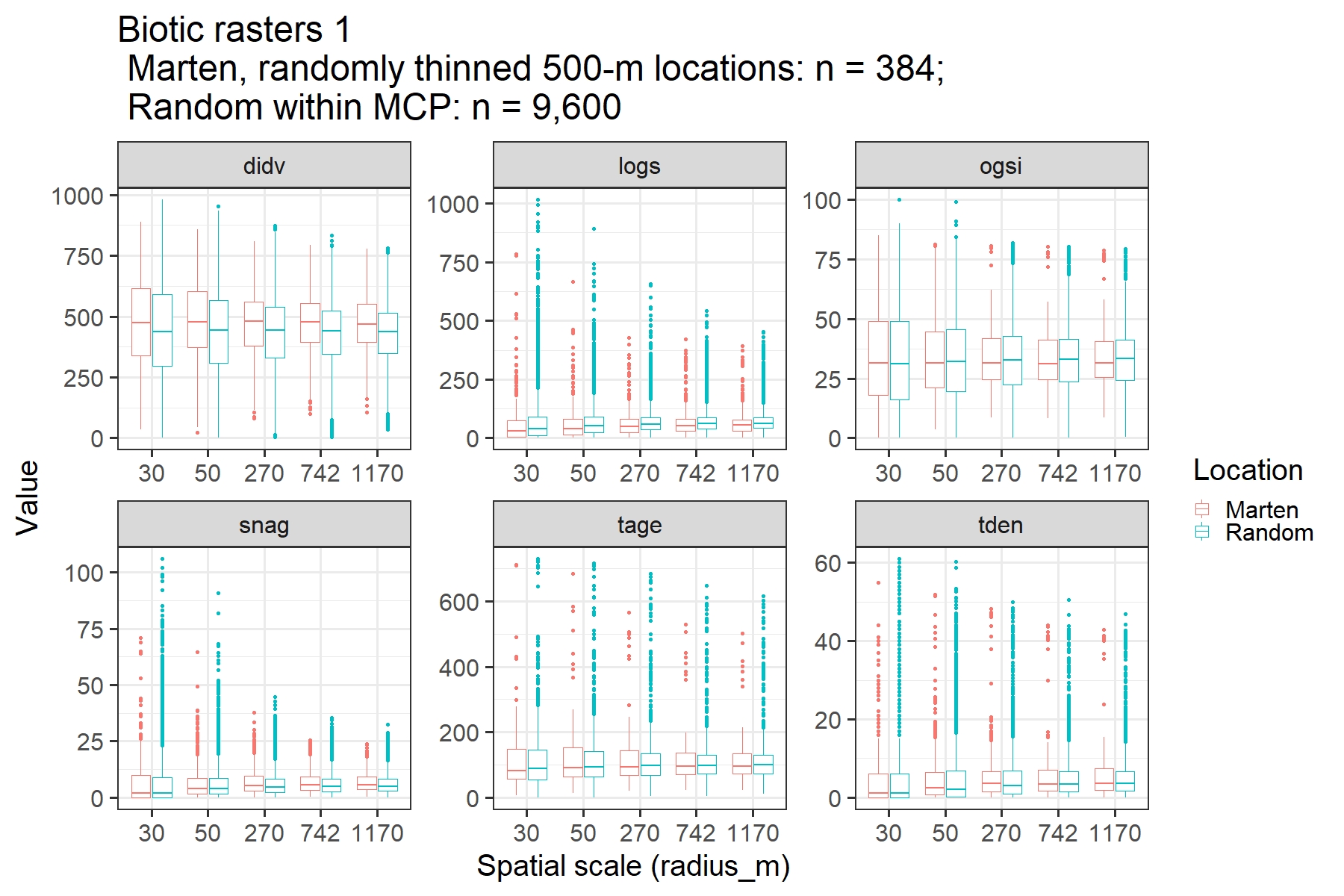

Supplement: Supplemental Information 5 — To provide the range of values observed in this study, we depict boxplots for the biotic variables in the top model showing the thinned marten data and random locations (25/marten location; 9,600 random locations) for each spatial scale (radius = 50 m, 270 m, 742 m, 1,170 m) associated with Humboldt marten biology (e.g., Table S1, described in methods). [file peerj-09-11670-s005.jpeg]

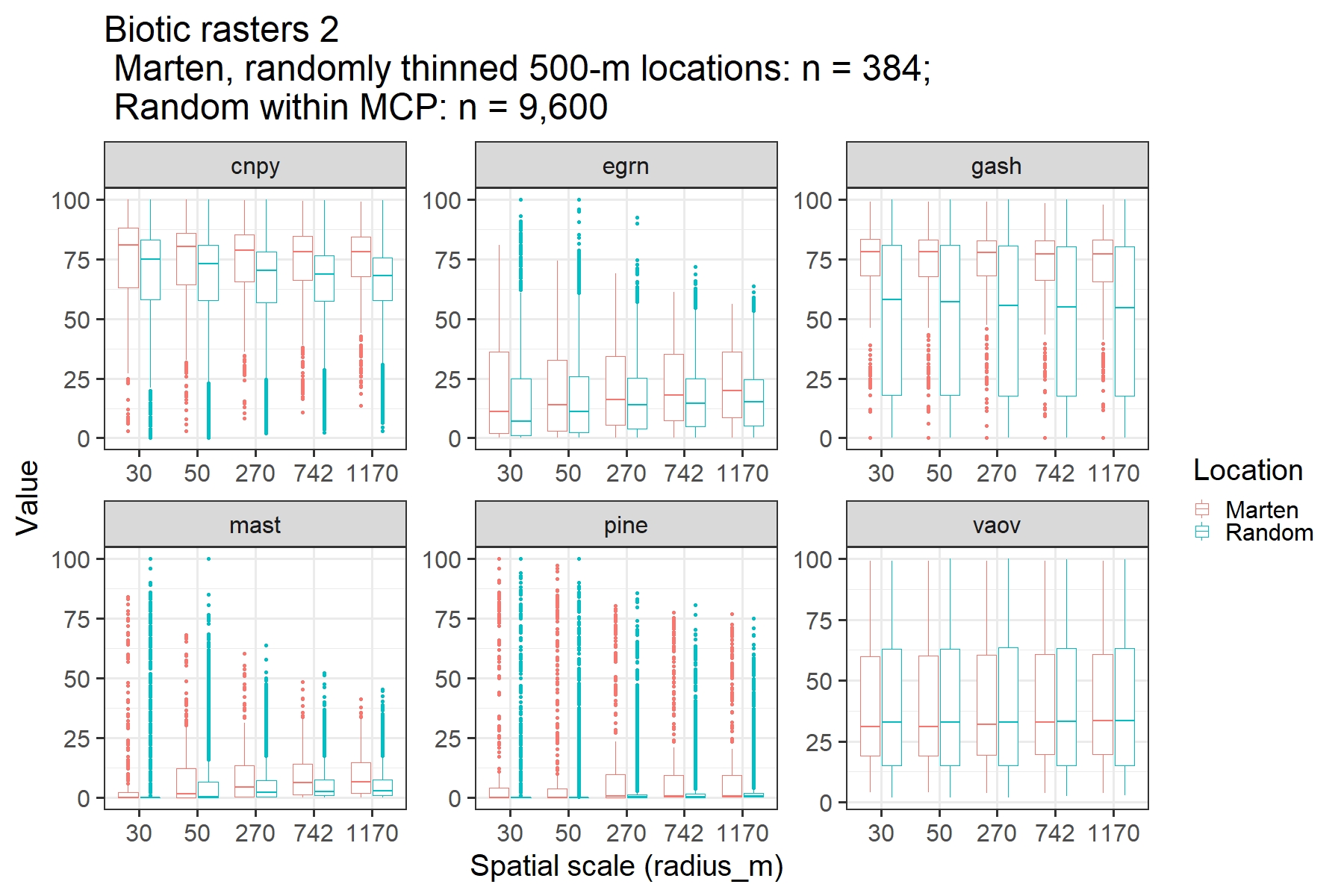

Supplement: Supplemental Information 6 — To provide the range of values observed in this study, we depict boxplots for the biotic variables in the top model showing the thinned marten data and random locations (25/marten location; 9,600 random locations) for each spatial scale (radius = 50 m, 270 m, 742 m, 1,170 m) associated with Humboldt marten biology (e.g., Table S1, described in methods). [file peerj-09-11670-s006.jpeg]

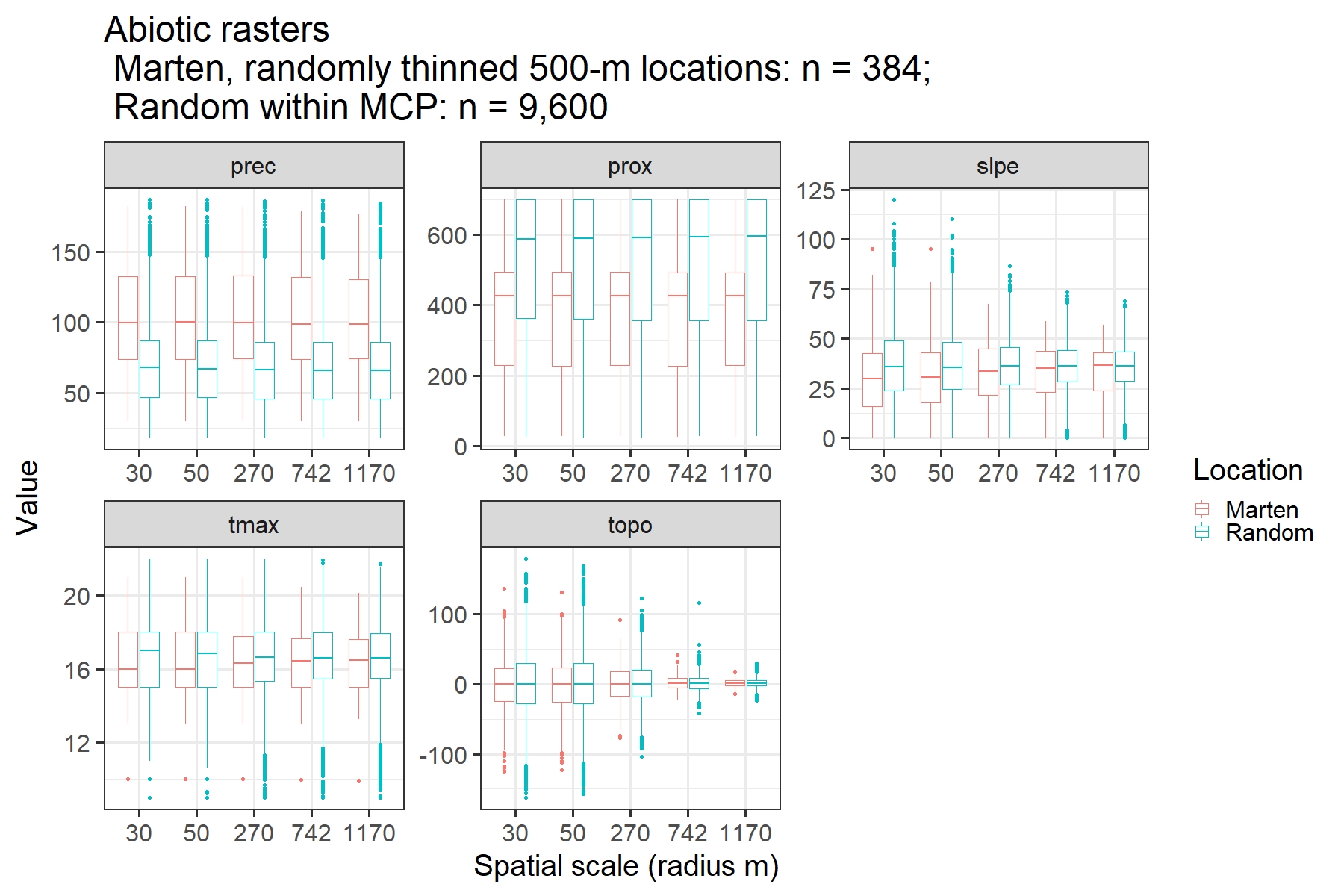

Supplement: Supplemental Information 7 — To provide the range of values observed in this study, we depict boxplots for the abiotic variables in the top model showing the thinned marten data and random locations (25/marten location; 9,600 random locations) for each spatial scale (radius = 50 m, 270 m, 742 m, 1,170 m) associated with Humboldt marten biology (e.g., Table S1, described in methods). [file peerj-09-11670-s007.jpeg]

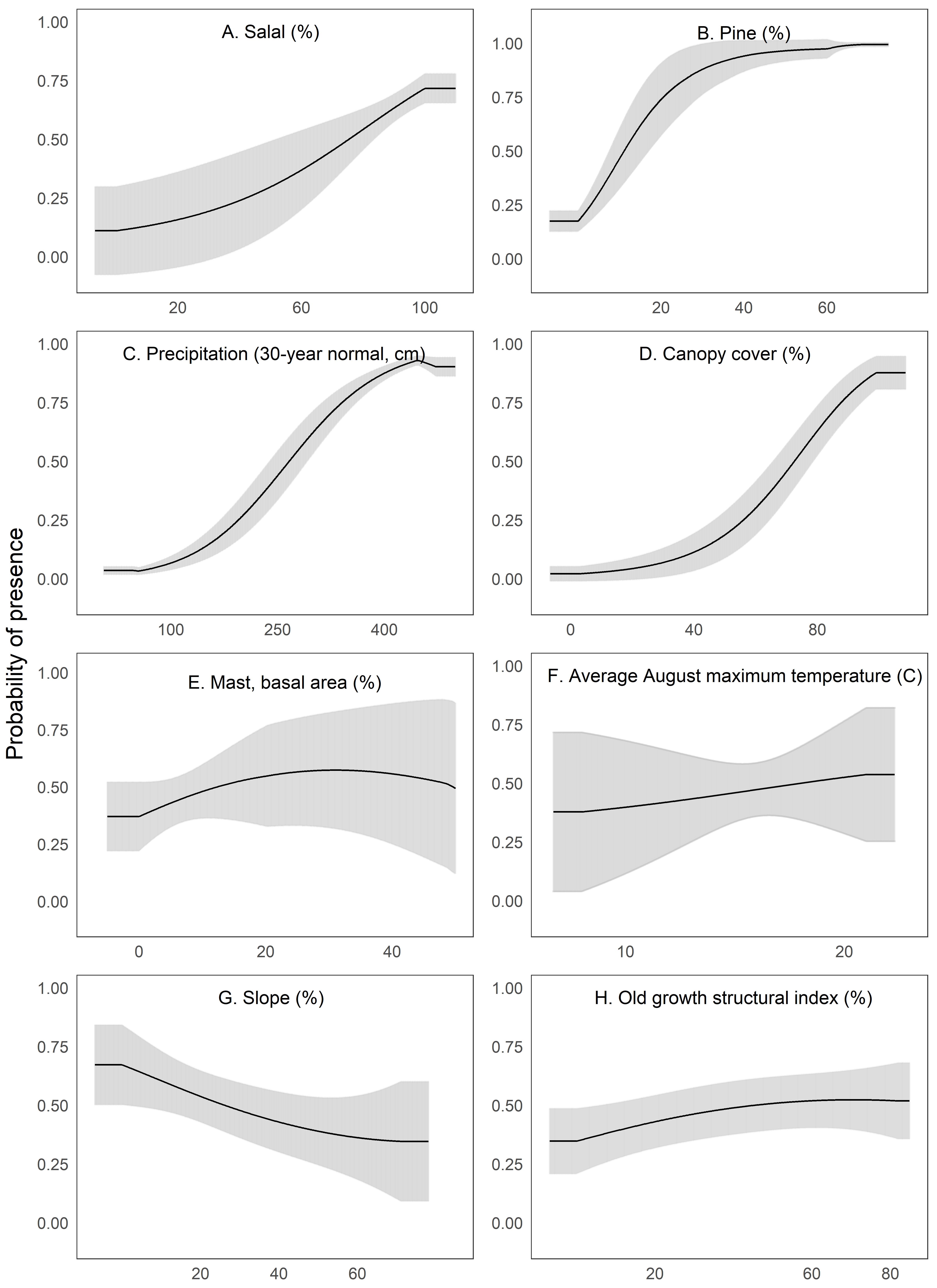

Supplement: Supplemental Information 8 — Here, each curve is the predicted probability of presence given the other variable responses. Predicted distribution of Humboldt martens were correlated with increasing canopy cover, percent pine, precipitation, temperature, mast, percent salal (Gaultheria shallon) distribution, and the old growth structural index.We observed a negative correlation between marten locations percent slope. Percent contribution and permutation importance values were reported in Table 2. The curves reveal the mean response (black) and one standard deviation (gray) for 10 replicate Maxent runs. [file peerj-09-11670-s008.jpg]
