# Supplementary material for: Predicted distribution of a rare and understudied forest carnivore: Humboldt marten (Martes caurina humboldtensis)
Source: PeerJ. 2021 Jul 21;9:e11670. doi: 10.7717/peerj.11670 (PMC8354145; doi:10.7717/peerj.11670)
Supplement: Supplemental Information 10 — We evaluated Pearson correlation coefficients and restricted variables with correlation >0.6 to guide variables in a Humboldt marten (Martes caurina humboldtensis) distribution model. For our final model, we selected canopy cover over the diameter diversity index. We selected OGSI over tree age or diameter diversity index due to its use in prior Humboldt marten range wide models. We selected salal over huckleberry because of salal’s presumed structural use (Eriksson et al., 2019; Moriarty et al., 2019). [file peerj-09-11670-s010.docx]

**Supplemental Table S2:**

**We evaluated Pearson correlation coefficients for inclusion in our distribution model.**

We evaluated Pearson correlation coefficients and restricted variables with correlation >0.6 to guide variables in a Humboldt marten (*Martes caurina humboldtensis*) distribution model. For our final model, we selected canopy cover over the diameter diversity index. We selected OGSI over tree age or diameter diversity index due to its use in prior Humboldt marten range wide models. We selected salal over huckleberry because of salal’s presumed structural use (Eriksson et al. 2019, Moriarty et al. 2019).

|  | Forest Age_270 | Canopy cover_1170 | Coastal proximity_50 | Diameter diversity index_1170 | Downed wood_270 | Salal_1170 | Mast_1170 | OGSI_50 | Percent pine_1170 | Percent slope_1170 | Precipitation_30yr_1170 | Snag density_742 | Max Aug temperature_30yr_1170 | Topographic position index_270 | Tree density_1170 | Vaccinium_1170 |
| --- | --- | --- | --- | --- | --- | --- | --- | --- | --- | --- | --- | --- | --- | --- | --- | --- |
| Forest Age_270 | 1 | 0.21 | 0.31 | 0.64 | 0.27 | -0.15 | 0.12 | 0.72 | -0.02 | 0.35 | 0.16 | 0.55 | -0.08 | -0.04 | 0.70 | -0.36 |
| Canopy cover_1170 | 0.21 | 1 | -0.19 | 0.76 | 0.34 | 0.09 | 0.31 | 0.30 | -0.51 | 0.09 | 0.06 | -0.07 | 0.11 | -0.01 | 0.40 | 0.19 |
| Coastal proximity_50 | 0.31 | -0.19 | 1 | 0.07 | -0.30 | -0.75 | 0.03 | 0.22 | 0.00 | 0.34 | -0.41 | -0.05 | 0.21 | 0.00 | -0.13 | -0.84 |
| Diameter diversity index_1170 | 0.64 | 0.76 | 0.07 | 1 | 0.36 | -0.05 | 0.24 | 0.60 | -0.32 | 0.29 | 0.12 | 0.36 | -0.05 | 0.00 | 0.74 | -0.11 |
| Downed wood_270 | 0.27 | 0.34 | -0.30 | 0.36 | 1 | 0.29 | -0.14 | 0.36 | -0.19 | 0.00 | 0.05 | 0.39 | -0.26 | -0.03 | 0.49 | 0.30 |
| Salal_1170 | -0.15 | 0.09 | -0.75 | -0.05 | 0.29 | 1 | -0.09 | -0.16 | 0.01 | -0.10 | 0.65 | 0.16 | -0.34 | 0.01 | 0.25 | 0.79 |
| Mast_1170 | 0.12 | 0.31 | 0.03 | 0.24 | -0.14 | -0.09 | 1 | 0.10 | -0.10 | 0.30 | 0.25 | -0.13 | 0.47 | -0.03 | 0.06 | -0.15 |
| OGSI_50 | 0.72 | 0.30 | 0.22 | 0.60 | 0.36 | -0.16 | 0.10 | 1 | -0.12 | 0.27 | 0.03 | 0.44 | -0.08 | -0.04 | 0.51 | -0.26 |
| Percent pine_1170 | -0.02 | -0.51 | 0.00 | -0.32 | -0.19 | 0.01 | -0.10 | -0.12 | 1 | -0.13 | 0.11 | 0.17 | -0.10 | 0.01 | -0.16 | -0.05 |
| Percent slope_1170 | 0.35 | 0.09 | 0.34 | 0.29 | 0.00 | -0.10 | 0.30 | 0.27 | -0.13 | 1 | 0.18 | 0.23 | -0.01 | -0.01 | 0.23 | -0.32 |
| Precipitation_30yr_1170 | 0.16 | 0.06 | -0.41 | 0.12 | 0.05 | 0.65 | 0.25 | 0.03 | 0.11 | 0.18 | 1 | 0.32 | -0.19 | 0.00 | 0.32 | 0.26 |
| Snag density_742 | 0.55 | -0.07 | -0.05 | 0.36 | 0.39 | 0.16 | -0.13 | 0.44 | 0.17 | 0.23 | 0.32 | 1 | -0.48 | 0.03 | 0.61 | -0.06 |
| Max Aug temperature_30yr_1170 | -0.08 | 0.11 | 0.21 | -0.05 | -0.26 | -0.34 | 0.47 | -0.08 | -0.10 | -0.01 | -0.19 | -0.48 | 1 | -0.06 | -0.16 | -0.21 |
| Topographic position index_270 | -0.04 | -0.01 | 0.00 | 0.00 | -0.03 | 0.01 | -0.03 | -0.04 | 0.01 | -0.01 | 0.00 | 0.03 | -0.06 | 1 | 0.00 | 0.01 |
| Tree density_1170 | 0.70 | 0.40 | -0.13 | 0.74 | 0.49 | 0.25 | 0.06 | 0.51 | -0.16 | 0.23 | 0.32 | 0.61 | -0.16 | 0.00 | 1 | 0.09 |
| Vaccinium_1170 | -0.36 | 0.19 | -0.84 | -0.11 | 0.30 | 0.79 | -0.15 | -0.26 | -0.05 | -0.32 | 0.26 | -0.06 | -0.21 | 0.01 | 0.09 | 1 |
