# Supplementary material for: Predicted distribution of a rare and understudied forest carnivore: Humboldt marten (Martes caurina humboldtensis)
Source: PeerJ. 2021 Jul 21;9:e11670. doi: 10.7717/peerj.11670 (PMC8354145; doi:10.7717/peerj.11670)
Supplement: Supplemental Information 11 — The absolute value of variance inflation factors equal to 1 are considered not correlated and values greater than 5 are highly correlated as determined by Velleman & Welsch (1981). Here, diameter diversity index conflicted with canopy cover and we used canopy cover in our final model for the ease of interpretation and use in a management context. [file peerj-09-11670-s011.docx]

**Supplemental Table S3:**

**We evaluated Variance Inflation Factors, which reveal correlation and multicollinearity, to guide variables in a Humboldt marten (*Martes caurina humboldtensis*) distribution model.**

The absolute value of variance inflation factors equal to 1 are considered not correlated and values greater than 5 are highly correlated as determined by Velleman & Welsch (1981). Here, diameter diversity index conflicted with canopy cover and we used canopy cover in our final model for the ease of interpretation and use in a management context.

|  | Forest Age_270 | Canopy cover_1170 | Coastal proximity_50 | Diameter diversity index_1170 | Downed wood_270 | Salal_1170 | Mast_1170 | OGSI_50 | Percent pine_1170 | Percent slope_1170 | Precipitation_30yr_1170 | Snag density_742 | Max Aug temperature_30yr_1170 | Topographic position index_270 | Tree density_1170 | Vaccinium_1170 |
| --- | --- | --- | --- | --- | --- | --- | --- | --- | --- | --- | --- | --- | --- | --- | --- | --- |
| Forest Age_270 | 4.49 | 0.13 | -1.10 | -0.22 | 0.01 | 0.33 | -0.06 | -1.59 | -0.32 | 0.06 | -0.60 | -0.22 | 0.04 | 0.11 | -2.21 | 0.30 |
| Canopy cover_1170 | 0.13 | 6.91 | 0.72 | -6.99 | -1.01 | 0.17 | -0.50 | 0.29 | 1.16 | 0.14 | -0.24 | 1.43 | -0.36 | -0.04 | 2.17 | -1.33 |
| Coastal proximity_50 | -1.10 | 0.72 | 4.92 | -0.62 | 0.33 | 0.08 | 0.51 | 0.12 | 0.19 | -0.87 | 1.02 | 0.54 | -0.17 | -0.04 | 0.60 | 2.88 |
| Diameter diversity index_1170 | -0.22 | -6.99 | -0.62 | 10.65 | 1.03 | 0.85 | -0.16 | -1.08 | -0.65 | -0.23 | 0.14 | -0.82 | 0.98 | 0.00 | -4.73 | 1.11 |
| Downed wood_270 | 0.01 | -1.01 | 0.33 | 1.03 | 1.98 | -0.46 | 0.12 | -0.51 | 0.06 | -0.03 | 0.66 | -0.58 | 0.12 | 0.06 | -0.76 | 0.12 |
| Salal_1170 | 0.33 | 0.17 | 0.08 | 0.85 | -0.46 | 7.55 | 0.55 | 0.04 | 0.17 | -0.40 | -3.66 | 0.59 | 0.68 | -0.02 | -1.19 | -4.36 |
| Mast_1170 | -0.06 | -0.50 | 0.51 | -0.16 | 0.12 | 0.55 | 2.04 | -0.04 | -0.11 | -0.53 | -0.82 | 0.01 | -0.90 | -0.01 | 0.34 | 0.13 |
| OGSI_50 | -1.59 | 0.29 | 0.12 | -1.08 | -0.51 | 0.04 | -0.04 | 2.54 | 0.14 | -0.02 | 0.14 | -0.30 | -0.10 | 0.02 | 0.92 | -0.02 |
| Percent pine_1170 | -0.32 | 1.16 | 0.19 | -0.65 | 0.06 | 0.17 | -0.11 | 0.14 | 1.55 | 0.22 | -0.19 | -0.25 | -0.05 | -0.01 | 0.55 | -0.27 |
| Percent slope_1170 | 0.06 | 0.14 | -0.87 | -0.23 | -0.03 | -0.40 | -0.53 | -0.02 | 0.22 | 1.59 | -0.17 | -0.22 | 0.18 | 0.01 | 0.00 | 0.07 |
| Precipitation_30yr_1170 | -0.60 | -0.24 | 1.02 | 0.14 | 0.66 | -3.66 | -0.82 | 0.14 | -0.19 | -0.17 | 3.70 | -0.56 | 0.02 | 0.03 | 0.07 | 2.25 |
| Snag density_742 | -0.22 | 1.43 | 0.54 | -0.82 | -0.58 | 0.59 | 0.01 | -0.30 | -0.25 | -0.22 | -0.56 | 3.18 | 0.95 | -0.05 | -1.06 | 0.18 |
| Max Aug temperature_30yr_1170 | 0.04 | -0.36 | -0.17 | 0.98 | 0.12 | 0.68 | -0.90 | -0.10 | -0.05 | 0.18 | 0.02 | 0.95 | 2.09 | 0.06 | -1.04 | -0.04 |
| Topographic position index_270 | 0.11 | -0.04 | -0.04 | 0.00 | 0.06 | -0.02 | -0.01 | 0.02 | -0.01 | 0.01 | 0.03 | -0.05 | 0.06 | 1.01 | -0.08 | 0.03 |
| Tree density_1170 | -2.21 | 2.17 | 0.60 | -4.73 | -0.76 | -1.19 | 0.34 | 0.92 | 0.55 | 0.00 | 0.07 | -1.06 | -1.04 | -0.08 | 6.03 | -0.59 |
| Vaccinium_1170 | 0.30 | -1.33 | 2.88 | 1.11 | 0.12 | -4.36 | 0.13 | -0.02 | -0.27 | 0.07 | 2.25 | 0.18 | -0.04 | 0.03 | -0.59 | 6.79 |
